# Possible atypical heparin-induced thrombocytopenia presenting with splenic infarction despite preserved platelet counts after cardiac surgery – a case report

**DOI:** 10.3389/fmed.2026.1867949

**Published:** 2026-07-29

**Authors:** Thomas M. Hofbauer, Max Lenz, Patrick Haider, Daniel Zimpfer, Cihan Ay, Peter Quehenberger, Bernhard Richter, Robert Zilberszac

**Affiliations:** 1 Department of Cardiology, Internal Medicine II, Medical University of Vienna, Vienna, Austria; 2 Department of Cardiac and Thoracic Aortic Surgery, Medical University of Vienna, Vienna, Austria; 3 Division of Hematology and Hemostaseology, Department of Medicine I, Medical University of Vienna, Vienna, Austria; 4 Department of Laboratory Medicine, Medical University of Vienna, Vienna, Austria

**Keywords:** 4T score, cardiopulmonary bypass, case report, heparin-induced thrombocytopenia, splenic infarction

## Abstract

Heparin-induced thrombocytopenia (HIT) type 2 is a major complication of heparin therapy and associated with considerable morbidity and mortality. Correct diagnosis, especially in the intensive care setting where thrombocytopenia occurs frequently, is of paramount importance. We report a 44-year-old female patient undergoing surgical replacement of the tricuspid valve due to Ebstein anomaly. Peri-operatively, the patient was treated with unfractionated heparin (UFH). Sixteen days after treatment initiation, computed tomography incidentally revealed multiple splenic infarctions consistent with embolic infarction. The 4T score was 3 points, suggesting a low pre-test probability for HIT; however, its applicability was limited by post-cardiac surgery platelet kinetics and the absence of a typical platelet fall. A PF4/heparin antibody immunoassay was therefore performed and showed strong positivity (optical density 2.07, lower limit of normal <0.51). Anticoagulation was changed to argatroban, followed by apixaban. Importantly, although an initial perioperative platelet decline of 59% was observed consistent with cardiopulmonary bypass-related consumption, platelet counts had recovered to within the normal range at the time of diagnosis, and no classical secondary platelet fall was observed. In summary, we report a case of possible HIT in which platelet count-based risk stratification may not have fully captured an atypical presentation in this selected clinical setting.

## Introduction

In the intensive care setting, both unfractionated heparin (UFH) and low-molecular-weight heparin (LMWH) are used for prophylactic or therapeutic anticoagulation. While administration is generally safe, bleeding is a frequent complication. Heparin-induced thrombocytopenia (HIT), another major complication related to administration of heparin, manifests in two forms (1): type 1 is a transient, non-immunological form occurring within one to 4 days of exposure. Importantly, type 1 HIT is not associated with thrombosis. Conversely, type 2 HIT is defined as formation of IgG antibodies against platelet factor 4 (PF4) bound to heparin, leading to potentially life-threatening thrombocytopenia and arterial and/or venous thrombosis (1). Both morbidity and mortality of HIT are considerable (2). In a large meta-analysis of 15 studies, the risk of HIT was reported as 2.6% for UFH and 0.2% for LMWH, respectively (3).

Correct diagnosis of HIT is paramount and commonly made via the 4T score (4, 5). The 4T score comprises severity of thrombocytopenia, clinical signs of thrombosis, timing of onset after heparin exposure, and presence or absence of other causes of thrombocytopenia. The 4T score, if low, was shown to have an excellent negative predictive value, while an intermediate to high probability requires subsequent evaluation (6). Once suspected, discontinuation of heparin and switch to non-heparin-based anticoagulation is urgently indicated, followed by further laboratory analysis.

Especially in patients treated in an intensive care unit (ICU), thrombocytopenia occurs frequently, with a reported incidence ranging between 15% and 50% (7, 8), which complicates the diagnosis of HIT in this context. Furthermore, atypical presentations of HIT even without overt thrombocytopenia (i.e., a fall of platelets below the lower limit of normal of 150 G/L) have been reported but remain incompletely characterized (9).

We report a case of possible HIT in a patient without overt thrombocytopenia at the time of clinical manifestation, which was only suspected after computed tomography revealed multiple splenic infarctions despite ongoing heparin exposure.

## Case description

The patient (female, 44 years) was referred to the department of cardiac surgery due to progressive dyspnea on exertion in the setting of known Ebstein anomaly of the tricuspid valve. Other relevant known diseases included obesity (BMI 31.6), a history of prior smoking (total of six pack years), obstructive sleep apnea and hypothyroidism. There was no history of allergies. There was no history of heparin exposure within the previous 100 days.

Invasive hemodynamic measurement 6 months prior to surgery excluded pulmonary hypertension (mean pulmonary arterial pressure 19 mmHg, pulmonary arterial wedge pressure 9 mmHg). The right ventricle was moderately to severely dilated, with normal right ventricular function. Tricuspid valve regurgitation was, as expected, severe.

The patient underwent surgery under cardiopulmonary bypass (CPB) support. Since tricuspid valve reconstruction turned out not to be technically viable, biological valve replacement (33 mm, Edwards Mitris) was performed. Intra-operatively, a cumulative dose of 60,000 units of UFH was administered. Subsequently, the patient was transferred to the ICU for post-operative care, where she presented hemodynamically stable under minimal vasopressor support. Post-operative transthoracic echocardiography did not reveal residual tricuspid regurgitation or stenosis.

The patient was extubated on post-operative-day (POD)1. Only 1 h later, she developed acute hypoxemia and was thus promptly re-intubated. Computed tomography of the chest on POD1, POD10 and POD17, together with daily chest X-rays, indicated dorsal atelectasis, but no infectious pulmonary consolidations. Leukocyte count, levels of C-reactive protein and procalcitonin are given in Table 1. Empirical antimicrobial therapy was initiated with ampicillin/sulbactam, followed by meropenem, linezolid and ceftazidime/avibactam upon diagnosis of *Staphylococcus epidermidis* biofilm on a central line; no other bacteria, fungi or viruses could be cultivated. Infective endocarditis was ruled out using transesophageal echocardiography. Several proning cycles were initiated, and tracheostomy was performed on POD16, resulting in improvement of oxygenation and ventilation.

**TABLE 1:** Laboratory analyses.

Day	LEUK (G/L)	CRP (mg/dL)	PCT (ng/mL)	PLT (G/L)	aPTT (sec)	D-dimer (μg/mL)	Hemoclot (μg/mL)	Comment
**Pre**-OP	9.43	0.68	0.10	283	72.8			
**OP**	24.17	0.68	0.10	117				
**POD1**	18.36	5.09	0.10	140				UFH 300 IU/h
**POD2**	19.82	27.06	30.20	116	45.6			
**POD3**	20.34	30.64	26.40	130	47.2			
**POD4**	18.51	18.18	14.30	115	39.2			
**POD5**	16.62	9.72	7.99	147	36.6			
**POD6**	15.77	9.27	3.73	187	33.1			
**POD7**	16.31	11.88	1.98	208	34.8			
**POD8**	16.42	14.90	1.19	219	37.9			
**POD9**	13.46	18.60	0.80	235	39.6			
**POD10**	10.34	23.03	0.61	248	38.6			
**POD11**	9.73	19.18	0.46	284	40.4			
**POD12**	11.17	19.29	0.35	328	40.4			
**POD13**	11.10	20.40	0.33	315	41.4			
**POD14**	11.46	19.60	0.29	327	41.6			
**POD15**	9.78	19.10	0.29	321	39.2			
**POD16**	8.86	18.20	0.30	295	34.5			Tracheostomy
**POD17**	11.07	23.04	0.35	287	40.5	10.1		HIT, argatroban
**POD18**	11.14	22.4	0.40	274	59.3		0.19	
**POD19**	9.82	12.80	0.37	265	65.6	10.4	0.37	
**POD20**	10.08	9.96	0.26	257	53.0	11.8	0.28	
**POD21**	10.14	7.18	0.20	226	59.8	11.9	0.36	
**POD22**	10.74	6.75	0.16	242	57.8	12.2	0.45	
**POD23**	10.93	5.22	0.12	198	62.9	12.8	0.52	
**POD24**	10.95	3.96	0.10	213	58.8	11.4	0.57	
**POD25**	10.14	3.28	0.09	187	68.4	8.8	0.46	
**POD26**	9.87	5.15	0.09	193	67.9	8.1	0.58	
**POD27**	9.76	6.28	0.06	187	70.1	6.1	0.65	
**POD28**	12.27	4.25	0.05	165	63.3	5.5	0.60	
**POD29**	10.59	6.64	0.05	159	70.4	3.4	0.58	
**POD30**	11.78	8.37	0.06	156	39.8	5.1	<0.1	
**POD31**	11.49	14.60	0.10	151	75.3	3	0.54	
**POD32**	9.70	14.50	0.08	144	73.3	2.5	0.75	
**POD33**	8.24	11.10	0.06	173	69.1	2.6	0.56	
**POD34**	8.74	7.34	0.06	198	67.5	2.3	0.50	
**POD35**	9.73	6.05	0.05	280				Apixaban

Empty cells indicate missing values. Hemoclot refers to the direct thrombin inhibitor assay used for monitoring argatroban dose (therapeutic target range 0.5–0.8 μg/mL at our center). aPTT, activated partial thromboplastin time; CRP, C-reactive protein; HIT, heparin-induced thrombocytopenia; LEUK, leukocytes; PCT, procalcitonin; PLT, platelets; POD, post-operative day; UFH, unfractionated heparin.

**FIGURE 1:**
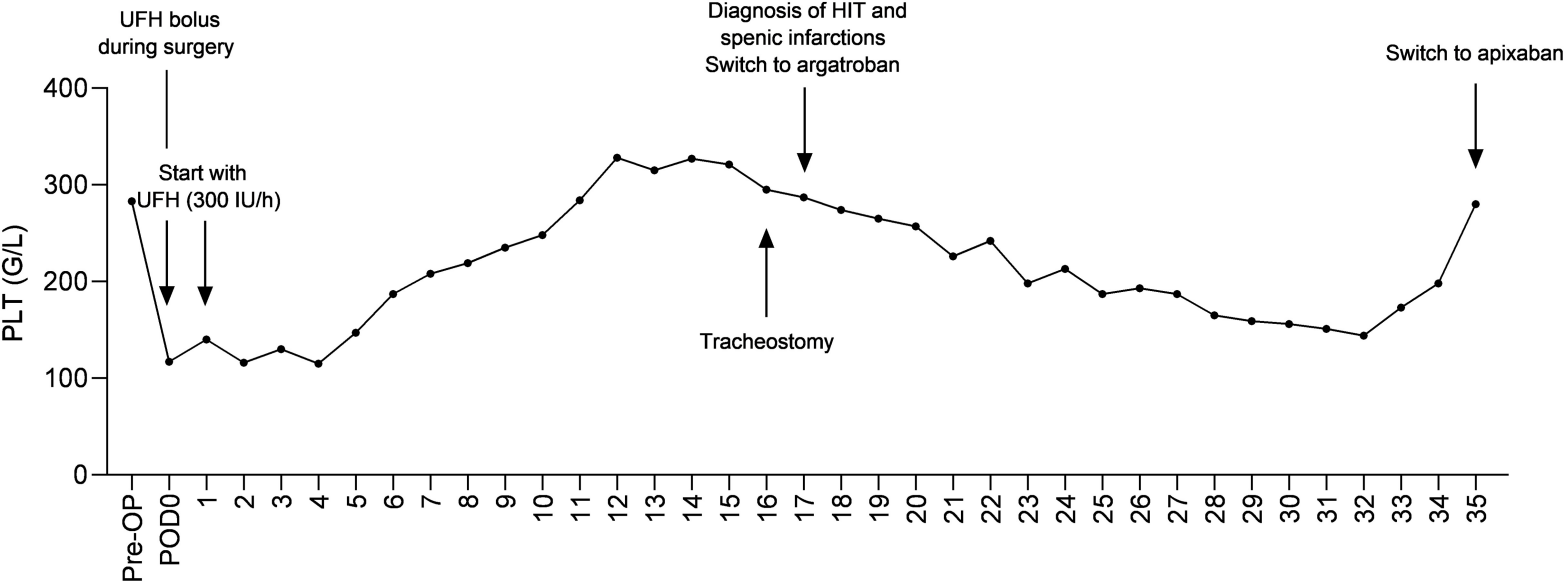
Trajectory of platelets during the course of treatment. HIT, heparin-induced thrombocytopenia; PLT, platelets; UFH, unfractionated heparin.

Unfractionated heparin was started on POD1, with a continuous infusion rate of 300 IE/h (fixed-dose prophylactic regimen in the absence of an indication for therapeutic anticoagulation). The trajectory of platelet count during hospitalization is given in Figure 1 and Table 1. Prior to surgery, the patient had a platelet count of 283 G/L (reference 162–379 G/L), indicating normal values. Upon admission to the ICU, platelets fell to 117 G/L. The lowest measured platelet count in this first phase occurred on POD4 (115 G/L), after which platelets rose steadily to 327 G/L on POD14.

**FIGURE 2:**
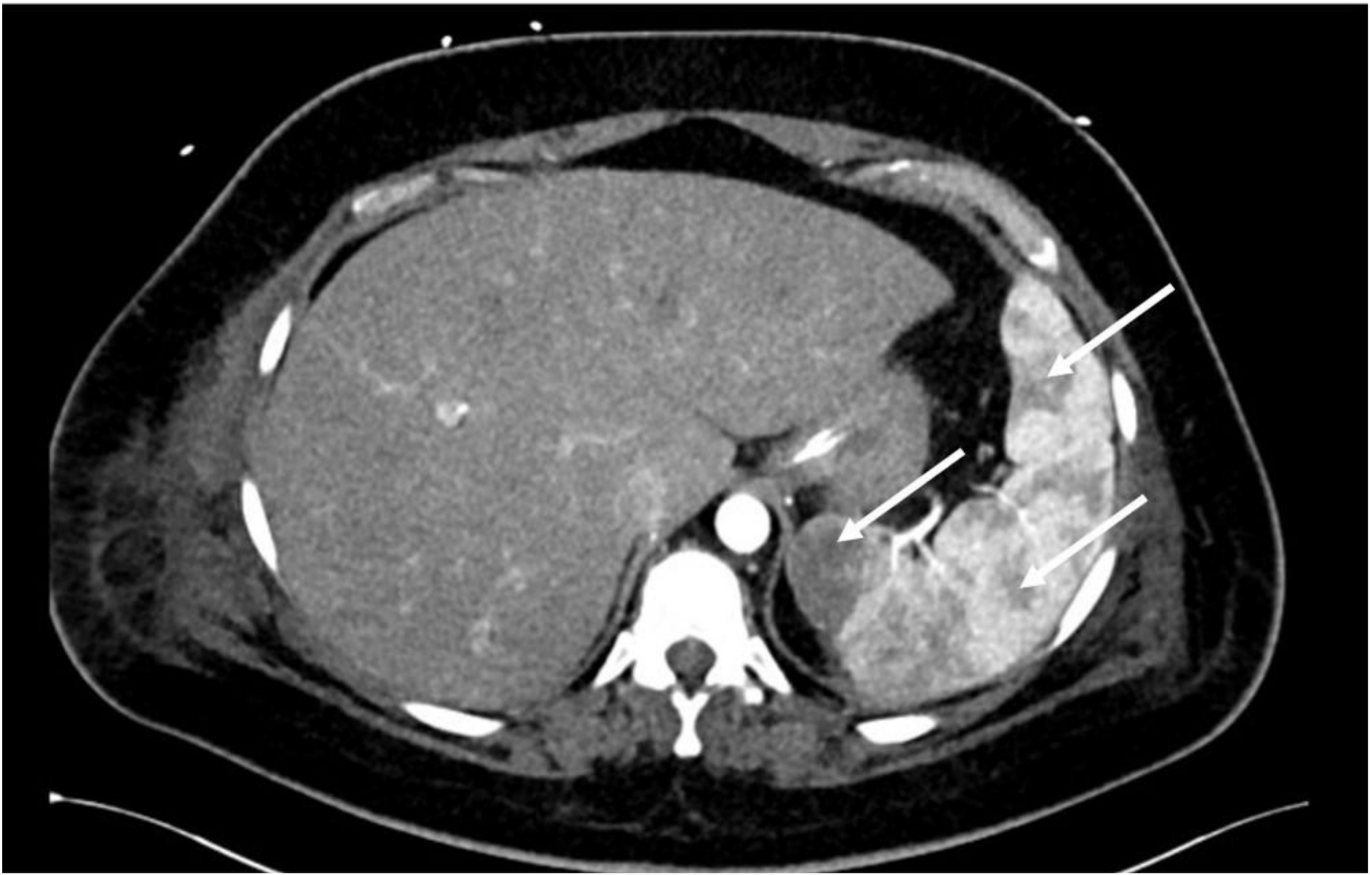
Computed tomography scan revealing splenic infarctions. Computed tomography of the abdomen on post-operative day 17 showed multiple splenic infarctions deemed to be of thromboembolic origin. White arrows indicate exemplary infarctions.

On POD17, due to protracted respiratory weaning, a CT scan of the lungs and abdomen was performed and unexpectedly revealed multiple splenic infarctions (affecting ∼20% of the parenchyma) consistent with embolic infarction. Importantly, no other organs were affected (Figure 2). That day, platelet count was 287 G/L (corresponding to a decline of ∼12% compared with POD14). Given the clinical suspicion of HIT, laboratory assessment revealed strongly positive PF4/heparin IgG antibodies (optical density 2.07, lower limit of normal <0.51, Zymutest HIA, Hyphen BioMed, Neuville-sur-Oise, France).

Anticoagulation was immediately switched from prophylactic-intensity heparin to therapeutic-intensity argatroban, a direct thrombin inhibitor, as recommended by international guidelines (5). D-dimer measurement on the day of diagnosis of heparin-induced thrombocytopenia (HIT) showed a markedly elevated level (10.1 μg/mL; upper limit of normal 0.5 μg/mL), which initially further increased and then declined steadily (Table 1). After switching from UFH to argatroban, no additional thrombotic events occurred. After 18 days of therapy with argatroban, the anticoagulation regimen was switched to oral apixaban 5 mg b.i.d. At 6-month telephone follow-up, the patient reported good clinical recovery and no clinically apparent recurrent thromboembolic events.

## Discussion

We report a case of possible atypical HIT, characterized by splenic infarction despite preserved platelet counts at the time of clinical manifestation. Furthermore, there was an absence of a clinically typical secondary platelet fall. Although an initial post-operative platelet decline was observed, consistent with CPB-related consumption, platelet counts had recovered to within the normal range at the time of diagnosis by a strongly positive anti-PF4/heparin IgG immunoassay, and no classical secondary decline was identified despite continued UFH exposure. Clinical suspicion of HIT arose only after detection of thromboembolic complications despite prophylactic heparinization. This unusual presentation highlights limitations of diagnostic heuristics that rely heavily on platelet count trends in the intensive care setting.

Heparin-induced thrombocytopenia classically presents with a platelet decline of >50%, typically occurring 5–10 days after exposure to heparin, and is associated with development of potentially debilitating thrombosis and thromboembolism (1, 10). Guidelines recommend using the 4T score, of which thrombocytopenia is one of four criteria (11). While the term HIT may suggest that absolute thrombocytopenia *per se* is mandatory for diagnosis, current consensus focuses on relative decreases in platelets rather than absolute platelet counts. With the unexpected discovery of multiple splenic infarctions on POD17, we suspected HIT and assessed the 4T score. There was no decrease in platelet count at that time, yielding 0 points; rather, platelets had steadily increased despite continued exposure to UFH. The timing of suspected HIT was longer than 10 days after initial exposure, yielding 1 point. Confirmed new thrombosis (i.e., splenic infarction) yielded 2 points. There were apparent alternative causes of thrombocytopenia (major cardiothoracic surgery, critical illness), yielding 0 points. Thus, the total 4T score was 3 points, corresponding to a low pre-test probability for HIT.

Guidelines advise against laboratory testing in patients with low clinical probability in typical presentations of HIT. However, given the atypical phenotype, ongoing heparin exposure and otherwise unexplained new thrombosis, we considered the conventional 4T framework insufficiently applicable in this context and proceeded to immunologic testing despite the formally low score. These limitations notwithstanding, the 4T score retains value as a structured clinical framework; as this case illustrates, clinical judgment remains essential when atypical thrombotic manifestations occur despite formally low scores.

Heparin-induced thrombocytopenia is intrinsically difficult to assess in critically ill patients due to a high incidence of thrombocytopenia resulting from other causes. Frequent causes are decreased production, destruction and consumption of platelets. This typically occurs in sepsis (8), but also in patients undergoing cardiac surgery (12) and cardiopulmonary bypass (CPB) (13). Thus, multiple factors confounding the platelet criterion of the 4T score exist in intensive care.

Final diagnosis of HIT relies on subsequent laboratory testing. As a first step, a serological immunoassay for anti-PF4/heparin IgG antibodies is recommended, followed by validation using a functional assay if the immunoassay is positive (5). In our patient, possible HIT was supported by a markedly positive PF4/heparin IgG immunoassay (optical density 2.07), together with new thromboembolism and ongoing heparin exposure despite the atypical platelet course.

Unfortunately, diagnosis of HIT suffers from relevant pitfalls: in healthy individuals, PF4/heparin antibodies can be detected in 1.0%–4.3% (14). Furthermore, it was demonstrated that about 50% of samples with detectable PF4/heparin antibodies do not contain platelet-activating antibodies (15). The issue of false positive results is even more accentuated in intensive care patients, where the false positive rate has been reported to be up to 80% (3, 16). Murine and human data suggest that glycosaminoglycans, bacterial antigens and bacterial DNA or RNA induce formation of PF4 antibodies, resembling HIT antigens recognized by conventional immunoassays (9). These factors predispose to overdiagnosis through false-positive immunoassays, while the complexity of platelet kinetics and competing causes for thrombocytopenia in ICU patients may also contribute to underrecognition of true HIT.

Our patient displayed several clinical features potentially skewing HIT diagnosis. Tricuspid valve replacement was performed with cardiopulmonary bypass (CPB) support. It has been shown that CPB itself is associated with development of PF4 antibodies in 22%–52% of cases, even in the absence of clinical HIT (11, 17). This may lead to false-positive results of immunoassay results and unnecessary changes in anticoagulation therapy. In patients after CPB, HIT typically follows a biphasic thrombocytopenic pattern, with a rapid drop immediately after surgery, and a second, delayed drop due to HIT itself (12). In the present case, platelet count fell by 59%, consistent with expected consumption due to CPB, followed by platelet recovery. However, a second, biphasic decline was not observed; instead, platelets paradoxically increased despite ongoing heparin exposure. This was especially true in the temporal window of 5–10 days after initiation of heparin, when HIT is clinically most apparent. Clinical manifestation was dominated by arterial thromboembolism (splenic infarction), suggesting that platelet activation and thrombosis can occur even in the absence of a classical biphasic platelet pattern.

The HIT immunoassay yielded a very high optical density. The probability of false-positive results decreases with stronger quantitative immunoassay positivity (18). We therefore did not pursue subsequent functional testing, a decision that may be supported by statistical modeling (5) based on three large analyses (6, 19, 20); nonetheless, the absence of functional confirmation (which is not available at our institution) remains an important limitation.

The markedly elevated D-dimer level in this case is consistent with the hypercoagulable state of HIT, although it is nonspecific in the ICU context. Since we did not expect thromboembolism, D-dimer was not assessed prior to diagnosis of HIT. As such, potential elevations after CPB might have been missed.

In the intensive care setting, several differential diagnoses for splenic infarction must be considered. A cardioembolic source appeared less likely because the patient did not develop transient perioperative atrial arrhythmias and did not undergo left-sided cardiac surgery. Furthermore, an intracardiac shunt or persistent foramen ovale, which could have enabled paradoxical embolization, had been excluded pre-operatively. Catheter-related thromboembolism was considered unlikely given the absence of supporting imaging findings. Low-flow states or hypoperfusion did not occur either during surgery or post-operatively. Non-HIT-related hypercoagulability and sepsis as an explanation for septic emboli were also considered unlikely, given the trajectory of leukocytes, CRP and PCT, and the absence of relevant systemic infection, as indicated by negative cultures. Splenic infarction is itself a rarely reported manifestation of HIT. The few reports available have predominantly described congestive, hemorrhagic splenic infarction or rupture, secondary to splenic vein thrombosis, analogous to bilateral adrenal hemorrhage attributed to adrenal vein thrombosis in the setting of HIT, rather than the arterial embolic infarction we observed in this case (21, 22). Arterial splenic infarction as a dominant presenting feature thus appears exceptional and further underscores the atypical nature of this presentation. Taken together, the combination of ongoing heparin exposure, new thrombosis and a strongly positive PF4/heparin immunoassay made HIT a likely underlying diagnosis.

This case highlights several considerations for clinical practice. First, HIT may become clinically apparent without overt thrombocytopenia, particularly when post-operative platelet kinetics obscure interpretation. Second, in ICU and post-cardiac surgery patients, platelet-based algorithms may be insufficient in isolation. Third, thromboembolic events may be the first or dominant clinical manifestation. Fourth, unexplained thrombosis during heparin exposure should prompt a low threshold for reconsidering HIT even when the formal pre-test score is low. Fifth, strongly positive immunoassays may carry substantial diagnostic weight when interpreted in the full clinical context. At the same time, this case should not be interpreted as an endorsement of liberal or reflexive serologic testing for HIT. Indiscriminate testing, especially in the critically ill and after cardiac surgery in whom PF4/heparin antibodies are common and the positive predictive value of the immunoassay is low, generates false positive results that prompt unnecessary changes to non-heparin anticoagulation, with corresponding bleeding risk and diagnostic confusion. In summary, considering HIT is warranted when a genuinely unusual or otherwise unexplained thrombosis develops after heparin exposure, even when platelet-based criteria are not met. Conversely, serologic testing should be discouraged in the absence of such features.

## Limitations

Causal inference is inherently limited by the nature of a single case report. We did not perform functional confirmation of HIT (e.g., serotonin release assay). While the combination of thrombosis, heparin exposure and strong immunoassay positivity suggests a high likelihood of HIT, the absence of functional confirmation precludes definitive diagnosis. This limitation is particularly relevant after cardiac surgery with cardiopulmonary bypass, in which PF4/heparin antibodies may occur in the absence of clinical HIT. Indeed, PF4/heparin antibodies have been detected in up to 50% of patients after CPB in the absence of clinical HIT, and false positive immunoassay rates of up to 80% have been reported in ICU patients; the strong quantitative positivity observed in this case reduces, but does not eliminate, this possibility. In addition, the splenic infarctions cannot be attributed to HIT with absolute certainty in retrospect. Because infarctions were asymptomatic and detected only on the first abdominal imaging obtained after surgery, their timing cannot be established with certainty. Accordingly, silent peri-operative systemic embolism related to CPB and surgical manipulation cannot be excluded as a contributing or alternative mechanism. Thus, this case is best interpreted as possible HIT within a complex clinical context. Importantly, the patient’s post-operative state, inflammation and/or infection may also have confounded interpretation of platelet trends. Finally, platelet counts did not recover promptly after substitution of heparin with argatroban but continued to decline over approximately 2 weeks before normalizing. Although platelet recovery is generally expected within days of heparin cessation in HIT, the trajectory of our patient possibly reflects ongoing consumption in the setting of persistent critical illness, constituting a further atypical feature.

## Conclusion

In summary, we present a case of possible atypical HIT after cardiac surgery. Clinically significant thrombosis may occur when post-operative platelet recovery masks the expected thrombocytopenic signal, and in the absence of a classical secondary platelet fall. In selected intensive care settings, platelet count-based screening may therefore underestimate the likelihood of HIT. Recognition of atypical phenotypes is important, since delayed diagnosis may prolong heparin exposure and ongoing thrombotic risk.

## Data Availability

The raw data supporting the conclusions of this article will be made available by the authors, without undue reservation.
